# Investigation on Task Shifting of HIV/AIDS Follow-Up Management Workers in New Launched Areas, China

**DOI:** 10.3390/ijerph15102132

**Published:** 2018-09-28

**Authors:** Rong Liu, Ling Huang, Qing Yang, Qiang Hu, Qing Huang, Xiaoqing Jiang, Hui Zhu, Fei Xie, Xiaotong Wen, Xiaojun Liu, Zhaokang Yuan, Yuanan Lu

**Affiliations:** 1 Jiangxi Province Key Laboratory of Preventive Medicine, School of Public Health, Nanchang University, Nanchang 330006, China; 416524116705@email.ncu.edu.cn (R.L.); huangling@email.ncu.edu.cn (L.H.); 406524215702@email.ncu.edu.cn (Q.H.); 406524216719@email.ncu.edu.cn (X.J.); 416524115699@email.ncu.edu.cn (H.Z.); 406530516742@email.ncu.edu.cn (F.X.); 406530517824@email.ncu.edu.cn (X.W.); 2 Centers for Disease Control and Prevention of Jiangxi Province, Nanchang 330000, China; yq024030@163.com (Q.Y.); huqiang1616@126.com (Q.H.); 3 Global Health Institute, Wuhan University, 115# Donghu Road, Wuhan 430071, China; 2017103050012@whu.edu.cn; 4 Department of Public Health Sciences, University of Hawaii at Mānoa, Honolulu, HI 96822, USA

**Keywords:** HIV/AIDS, task shifting, workers, attitudes, China

## Abstract

*Background*: This study aimed to understand attitudes of HIV/AIDS follow-up workers regarding task shifting, reveal the current conditions of this implementation, as well as to find out any challenges of early-stage implementation. *Methods*: Taking Jiangxi Province as an example, a cross-sectional survey with 102 health professionals in CDCs (Centers for Disease Control and Prevention) and 92 health care providers in primary health institutions was conducted from November 2016 to January 2017. This survey includes the demographic backgrounds of participants, their attitudes towards task shifting, and the main difficulties faced in their work, etc. *Results*: 60.8% of professionals and 77.2% of providers hold positive attitudes towards task shifting. Both health professionals and providers express their concerns about unclear and undefined funds distribution and lack of confidentiality of PLWHA (people living with HIV) in local primary health institutions. *Conclusions*: The majority of health workers hold positive attitudes towards task shifting. It also highlights some negative reactions in implementation, and reveals the main difficulties that constitute barriers to follow-up. Findings from this study may provide evidence for enhancing future implementation of task shifting.

## 1. Introduction

Human immunodeficiency virus (HIV)/acquired immune deficiency syndrome (AIDS) is a chronic infection that has affected more than 780 thousand people in China [1]. The impact of HIV and AIDS has compelled the Chinese government to implement approaches that will curb the spread of the virus and enhance care for those affected. The Chinese government officially released in 2006 the first *AIDS Prevention and Control Regulations* as the policy document guiding HIV/AIDS prevention and control [2]. It provides follow-up services for people living with HIV (PLWHA) in order to improve their quality of life, allow for better follow-up intervention and referral, and reduce secondary generation transmission, consequently becoming an essential part of AIDS prevention and treatment. Current evidence from other countries shows that antiretroviral treatment can prolong the PLWHA’s lives [3,4]. In China, the National Free Antiretroviral Treatment Program (NFATP), initiated in 2003, has significantly reduced the mortality of HIV patients [5]. Moreover, effective follow-up and local support can increase the use of antiviral treatment [6] and prevent high-risk behaviors [7,8]. Previous studies revealed that many areas lacked longer-term follow up of treatment monitoring [9], but Chinese PLWHA had access to increased lifelong follow-up services including treatment monitoring (i.e., CD4 cells and viral load measurement), medical counseling, referrals and behavioral interventions, spousal/fixed sex partners, and tuberculosis screening [10]. The conventional follow-up services have been carried out by Centers for Disease Control and Prevention (CDCs), which may remain a major limitation to achieving the goal of universal access to HIV services [11]. Additionally, the increasing number of PLWHA and the shortage of human resources are posing major challenges to the CDCs follow-up model [12]. To provide more convenient access for PLWHA, to cope with the shortage of professionals in CDCs, and to improve the operation efficiency of primary health institutions, China has started implementing task shifting for the scale-up of HIV follow-up management and prevention services [13]. The first implementation area was in Yunnan province in 2002 [14]. After decades, many areas such as Sichuan and Guangxi provinces have successfully employed this model to manage follow-up services.

Task shifting involved shifting follow-up services from CDCs to community health services centers or township hospitals. After expansion and decentralization of HIV/AIDS services to the primary care level, an increase in access to services with good outcomes was observed. There is evidence that task shifting also yields cost-savings and improves the efficiency of the health system [15,16]. However, there may be some new challenges preventing successful task shifting, especially in new areas.

In terms of HIV prevalence, Jiangxi Province ranks the 18th among all provinces in China [17]. Since 2015, the Finance Department of Jiangxi Province has allocated over 30 million Yuan to AIDS management programs for free HIV testing, treatment, and follow-up health care [18]. At the same time, the CDC (Center for Disease Control and Prevention) of Jiangxi Province has gradually piloted task shifting of follow-up management, which used to be carried out by workers from municipal, district, or county CDCs, to primary health institutions. This study is designed to take Jiangxi Province as an example to evaluate the current status of task shifting and examine attitudes of follow-up management workers from both CDCs and primary health institutions. Exploring health workers’ attitudes toward task shifting is an important issue when it comes to asking the participants “if they support the implementation of task shifting of HIV/AIDS follow-up management in their district?” Their attitude is often a determinant and predictor for the success of this program. Findings from this study could provide baseline information for improving the implementation of task shifting as well as AIDS prevention and control capabilities in other regions.

## 2. Methods and Materials

### 2.1. Study Setting

Jiangxi Province is situated in southeast China, spanning from the banks of the Yangtze River in the north to hillier areas in the south and east. Jiangxi is divided into 11 prefecture-level divisions and further subdivided into 100 county-level divisions. In recent years, the prevalence of AIDS has been continuously increasing [19], directly leading to a shortage of health professionals in CDCs.

### 2.2. Participants

Participants in this study involved two types of workers: (1) Health professionals in CDCs, in which 1 or 2 participants from all 100 counties were randomly selected. Professionals are referred to as workers who have been working in AIDS prevention and control in CDCs. Their roles in the task shifting process are to delegate partial work of follow-up management to primary health institutions (PHIs), to plan and organize this process before delegating, and to provide follow-up training, evaluations and supervision. (2) Health care providers are those selected from PHIs who have implemented task shifting. They are usually responsible for public health work assigned according to specific guidelines by the Chinese government, but are not professional in AIDs prevention [20]. Their roles in task shifting processes are to take over the work mentioned above from CDCs and to provide related follow-up services for PLWHA.

Based on the *Guidelines for Follow-up of People Living with HIV* released by the China CDC and the document released by the Jiangxi government [21], the contents of task shifting of follow-up management are listed in Table 1.

### 2.3. Questionnaires

A cross-sectional survey was conducted in this study, surveying a group of workers involved in HIV/AIDS follow-up management that included both professionals and providers by face-to-face interviews. With the assistance from the Jiangxi provincial CDC and local health officers, the survey was conducted between November 2016 and January 2017 by trained graduate students from Nanchang University, ensuring the quality of the data and avoiding any potential bias. The interview was conducted in an isolated room of local office areas to ensure privacy. Data was collected through the structured questionnaire. The questionnaire covers the demographic backgrounds of participants (gender, age, educational level, professional title, etc.), their attitudes towards task shifting and their reasoning behind their attitudes (by asking whether they are supporting task shifting or not and reasons), the main difficulties in their follow up work, etc.

### 2.4. Statistical Analysis

EpiData3.1 (EpiData Association, Odense, Denmark, http://epidata.dk) was used to set up the database. The IBM SPSS 19.0 (IBM Corporation, Chicago, IL, USA, http://www-01.ibm.com/software/analytics/SPSS/) statistical software package was applied to descriptive analysis and statistical data tests. Chi-square tests were applied to test the differences between professionals and providers. The significance level was set at *p* < 0.05.

## 3. Results

### 3.1. Demographics

The study was conducted with a total of 194 subjects, including 102 health professionals from CDCs and 92 health care providers from local PHIs (response rate 100%). These collected questionnaires were organized, numbered, and enrolled for data analysis. As shown in Table 2, 60.8% of professionals and 77.2% of providers hold positive attitudes towards task shifting. Majority of subjects lie between 25 and 54 years old (98.0% and 85.9% respectively) and their annual income mainly lies in RMB 30,000–49,999. As for professionals, 55.9% of them have a bachelor degree and 50.0% majored in preventive medicine. Of these professionals, 76.5% participants have worked for 2 to 9 years, and more than 90% are specialized in AIDS prevention and control. For providers, 42.4% of them have college degree, but only 19.6% with major in preventive medicine. Nearly 61% have 2 years or less of work experience, and none of them are specialized in AIDS prevention and control. In chi-square tests, there was no significant difference in sex, professional title and annual income between professionals and providers. In terms of age, education, profession, whether they specialize in AIDS prevention and control, their HIV/AIDS service years and attitudes towards to task shifting, the differences between professionals and providers are statistically significant (*p* < 0.05). Professionals’ education level is higher than the providers, there are more professionals than providers in preventive medicine, specializing in AIDS prevention, and they have generally been working for longer. However, the providers are more willing to accept task shifting than professionals.

### 3.2. The Reasons of Negative Attitudes towards Task-Shifting

As shown in Table 3, when asked why respondents don’t support task shifting, both health professionals and providers express their concerns about unclear and undefined fund distribution and lack of confidentiality of PLWHA in local PHIs. The professionals in CDCs also state that the nature of high turnover and lack of professional skills of primary health care providers is another concern. For health providers in PHIs, they claim that although they are already responsible for multiple tasks, it is hard for them to spend more special efforts on HIV/AIDS follow-up management work.

### 3.3. The Main Difficulties Faced by HIV/AIDS Follow-Up Management Workers

Table 4 summarizes the main difficulties faced by workers. For professionals, the main difficulties are heavy working pressure, little or no financial or material support, and low wages. But for providers, coordination problems with PLWHA are their main concern difficulties in follow-up work.

## 4. Discussion

Task shifting is a new strategy designed to help cope with current shortages of health workers [22]. This strategy has been successfully implemented in many countries and regions [23,24] as well as in some areas of China [25], and demonstrated that well-trained lay and community workers can successfully complete clearly defined tasks in both low- and high-income countries [26,27]. However, there might be challenges when strategically employing primary health providers as a means to improve follow up management in new launched areas. Previous studies concluded that task shifting could help improve access, coverage, and quality of HIV/AIDS related services only if other human resources, financial resources, health care quality issues, and the need for ongoing evaluation and research are addressed [28].

In this study, we found the average age of HIV/AIDS follow-up workers at primary levels in Jiangxi Province is equal with professionals working in CDCs [29]. There are statistical significantly differences between the professionals’ and providers’ educational level and major. Professionals are more educated than providers, especially in terms of college education. Half of them have majored in preventive medicine, but almost 80% of providers have no background in it. As we predicted, the overall quality of the primary levels are lower than CDCs, as primary health institutions are mostly employ lay workers. This is consistent in other areas as well [30]. As for annual income, both of them meet average levels compared with the 23,821 Yuan of national per capita income [31]. Most of the providers have worked in follow-up fields for less than 2 years. Even though Jiangxi Province has just started to implement task shifting follow-up management in 2015, there is a high turnover of providers. When it comes to whether they are specializing in AIDS prevention and control, their responses suggest a new problem in the primary care level. The overall providers are not full-time, follow-up workers and so they still have other duties and work to do.

One of the main purposes of this study is to evaluate the workers’ attitudes towards task shifting. This study shows that although the majority of participants hold positive attitudes, there are still some statistically significant differences between the participants. The providers are more willing to accept task shifting. Further exploration reveals that financial issues and confidentiality are their common concerns. Generally speaking, the accessibility and availability of sustainable financing is a major part of successful implementation of the strategy [32]. Funding for HIV health services are usually provided by the national or local governments. Even though there are some instructions on how to distribute these funds, the superior of the CDC and local administrators can make changes and distribution decision, which can directly influence their daily working environment and incomes of the participants. Thus, both professionals and providers considered it a negative attitude reason. Considerable apprehension exists in terms of the possible breaches in confidentiality with task shifting, the use of native health providers [33], as well as the substantial level of discrimination still existing significantly in China. This leads to a difference in the number of AIDS patients registered by the CDCs. Even if the health workers can provide follow-up services for AIDS patients, they are prone to loss of follow-up [34]. Thus, the health providers need to be constantly reminded of the effect that confidentiality breaches can have on the integrity of task shifting [35,36]. The professionals are responsible for supporting the implementation by providing support and skills training for providers, but the high turnover of providers could result in an increased work quantity for professionals. Moreover, some professionals expressed that it’s not necessary to compel the delegation because the current PLWHA is still under control. This echoes the guidelines by WHO indirectly, in that task shifting is just guidance for some overloaded areas, and it should be adapted to local conditions. The providers are in charging with several tasks and are not specialized in follow-up work. This condition can also be seen in other areas in China, making the manpower issues in primary medical levels still problematic.

The results from participants’ responses suggest that the higher authorities and related government agencies need to solve the difficulties in targeted areas, such as low wages. This is consistent with previous reports [37], suggesting that successful implementation of the program would need more financial support and effective distribution of the current funds. For professionals, there is a need to clearly define their work tasks [38], improve the working environment, increase income, and hire more workers so that it may help to relieve their work stress [39,40]. For providers, the professionals need to organize more professional training and guidelines that will help providers to learn how to communicate with PLWHA and improve follow-up work. Providers also need to get continuous in-service education and effective supervision, focus more on cognitive constructs-confidence, perseverance, knowledge, and medical care skills [41,42]. From this study it shows that the providers should focus more on the PLWHA, rather than themselves, since this is one of the reasons that the providers are more willing to accept task shifting. The implementation of task shifting needs appropriate financial remuneration to sustain the follow-up outputs and guarantee the workers’ enthusiasm [43].

## 5. Conclusions

This is the first study to evaluate follow-up management workers’ attitude towards task shifting and shows that the majority of workers hold positive attitudes to the program. However, this study also reveals several negative reasons affecting successful implementation of the program, including the main difficulties that constitute the barriers to follow-up. Findings from this study may provide evidence for local CDCs and other areas where task shifting is to be carried out, and help them to develop more effective approaches for fund-supporting and practical approaches to improve present follow-up work in future.

## 6. Limitations

This study only focuses on one province (Jiangxi) of China. Its limited sample areas may not reflect the overall situation in other regions in which the implementation of task shifting is in its early stages. In addition, the structured questionnaire may be a limited method of collecting data due to respondents only being able to answer the questions specifically asked and not gave independent opinions. This can also lead to the misclassification of true reasons behind negative attitudes. Finally, this survey aimed to understand workers’ attitudes towards task shifting, but we did not investigate and analyze the other influencing factors by statistical modeling. Further research is needed to explore the determinants of their attitudes.

## Figures and Tables

**Table 1:** The contents of HIV/AIDS follow-up management of workers.

Workers	Follow-Up Management Contents
Professionals	Collection and investigation of basic information Set up work network and develop personnel training Development of a plan of work Technical support and coordination for primary health institutions CD4 cell and viral load detection for HIV/AIDS Check and distribute the partial funds
Providers	The implementation of CDC issued work indicators Be responsible for informing HIV/AIDS of the CD4 and viral load results For those who meet the referral standard according to the requirements of the infected, carry out the corresponding referral work Complete spouse/fixed sex partner testing as required Complete case follow-up form filling and reporting To carry out publicity and education, consultation, behavioral intervention, condom distribution, psychological care, and support to the subjects followed up

**Table 2:** Socio-demographic characteristics of HIV/AIDS follow-up management workers *.

Variables	Professionals		Providers		χ^2^	*p*
	*N*	%	*N*	%		
Gender						
Male	53	52.0	52	56.5	0.41	0.52
Female	49	48.0	40	43.5		
Age (year)						
<25	2	2.0	4	4.3	12.43	0.00
25–54	100	98.0	79	85.9		
≥55	0	0.0	9	9.8		
Educational level						
High school graduate or below	12	11.8	26	28.3	17.57	0.00
Associate college	31	30.4	39	42.4		
College	57	55.9	27	29.3		
Master or above	2	2.0	0	0.0		
Major						
Preventive medicine	51	50.0	18	19.6	19.55	0.00
Others	51	50.0	74	80.4		
Professional title						
None	11	10.8	12	13.0	0.99	0.32
Primary	47	46.1	48	52.2		
Intermediate	41	40.2	29	31.5		
Senior	3	2.9	3	3.3		
Annual income (RMB)						
<10,000	2	2.0	0	0.0	0.17	0.68
10,000–29,999	26	25.5	29	31.52		
30,000–49,999	60	58.8	52	56.52		
≥50,000	14	13.7	11	11.96		
Specialized in AIDS **						
Yes	94	92.2	0	0	164.48	0.00
No	8	7.74	92	100.0		
HIV/AIDS service years (Year)						
<2	15	14.71	56	60.9	49.86	0.00
2–9	78	76.5	36	39.1		
≥10	9	8.8	0	0		
Attitude towards task-shifting						
Positive	62	60.8	71	77.2	6.03	0.02
Negative	40	39.2	21	22.8		

* *N* = frequency, % = percentage; ** specialized in AIDS Prevention and Control.

**Table 3:** The reasons of negative attitudes towards task shifting *.

Reasons	Professionals **		Providers ***	
	*N*	%	*N*	%
Unclear and undefined funds distribution	38	95.0	19	90.5
Lack of confidentiality of PLWHA in local primary health institutions	33	82.5	10	47.6
Lack of professional skills of providers	36	90.0	13	61.9
High turnover of providers	28	70.0	0	0
Acceptable number of PLWHA	25	62.5	0	0
Responsible for multiple works	0	0	21	100.0

* This is a multiple-choice question; ** *N* = 40; *** *N* = 21. PLWHA: people living with HIV.

**Table 4:** Main difficulties faced by HIV/AIDS follow-up management workers *.

Variables	Professionals **		Providers ***	
	*N*	%	*N*	%
Under heavy working pressure	72	70.6	23	25.0
Little or no financial or material support	67	65.7	26	28.3
Low income	62	60.8	29	31.5
Coordination problems with PLWHA	61	59.8	66	71.7
Lack of professional knowledge and skills	28	27.5	32	34.8
Under heavy psychological pressure	48	47.1	25	27.2
Unclear responsibilities	19	18.6	13	14.1

* This is a multiple-choice question; ** *N* = 102; *** *N* = 92.
